# Fascial Anatomy of the Thyroid Region: A Retropharyngeal Goiter Without Extension Into the Retropharyngeal Space

**DOI:** 10.1002/ccr3.72287

**Published:** 2026-03-10

**Authors:** Masami Suzuki, Naohiro Yoshida, Mikio Shimazaki

**Affiliations:** ^1^ Department of Otolaryngology‐Head and Neck Surgery Jichi Medical University Saitama Medical Center Saitama Japan

**Keywords:** cervical fascia, papillary thyroid carcinoma, retropharyngeal goiter, retropharyngeal space, visceral space

## Abstract

Intraoperative photographs in a patient with papillary thyroid carcinoma demonstrated that the non‐invasive retropharyngeal goiter did not extend into the retropharyngeal space.

## Key Clinical Message

This case provides intraoperative photographic evidence that a non‐invasive retropharyngeal goiter remains within the visceral space without extending into the retropharyngeal space, indicating that the middle layer of the deep cervical fascia functions as a barrier to such extension.

## 1 Case Presentation

**FIGURE 1:**
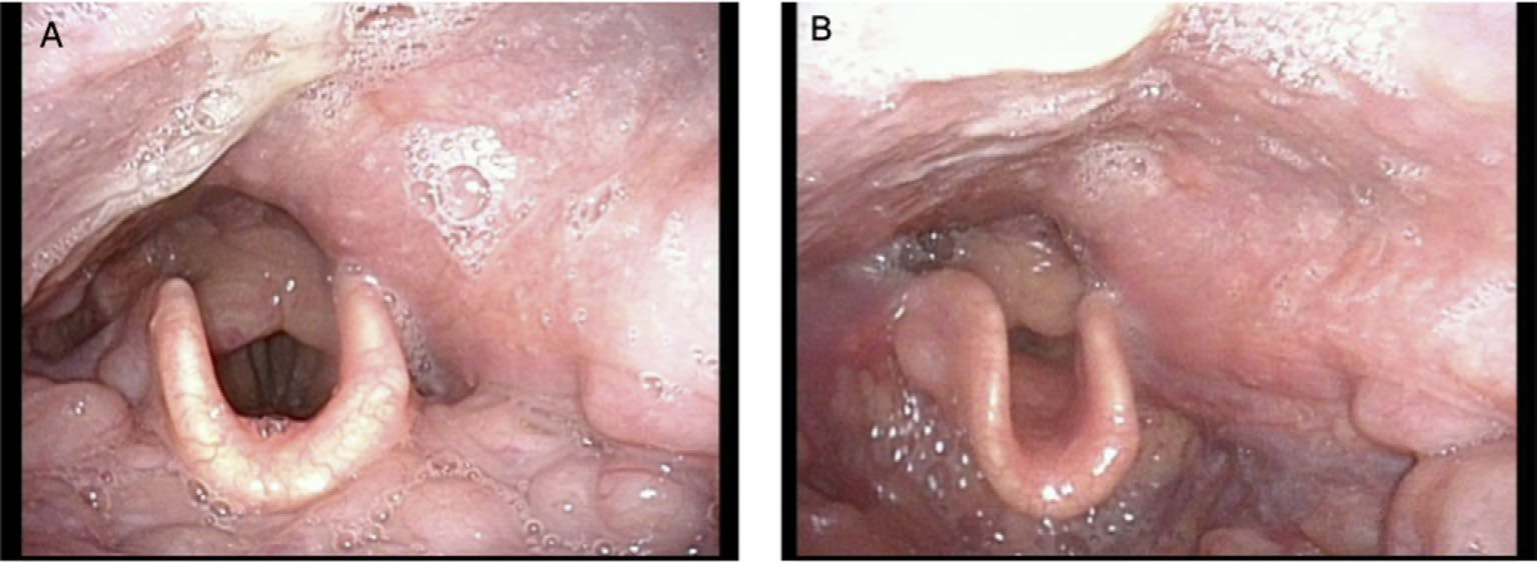
Laryngoscopic findings. (A) Larynx during inspiration. (B) Larynx during phonation. The left posterior and lateral walls of the pharynx were swollen. Vocal cord mobility was normal.

**FIGURE 2:**
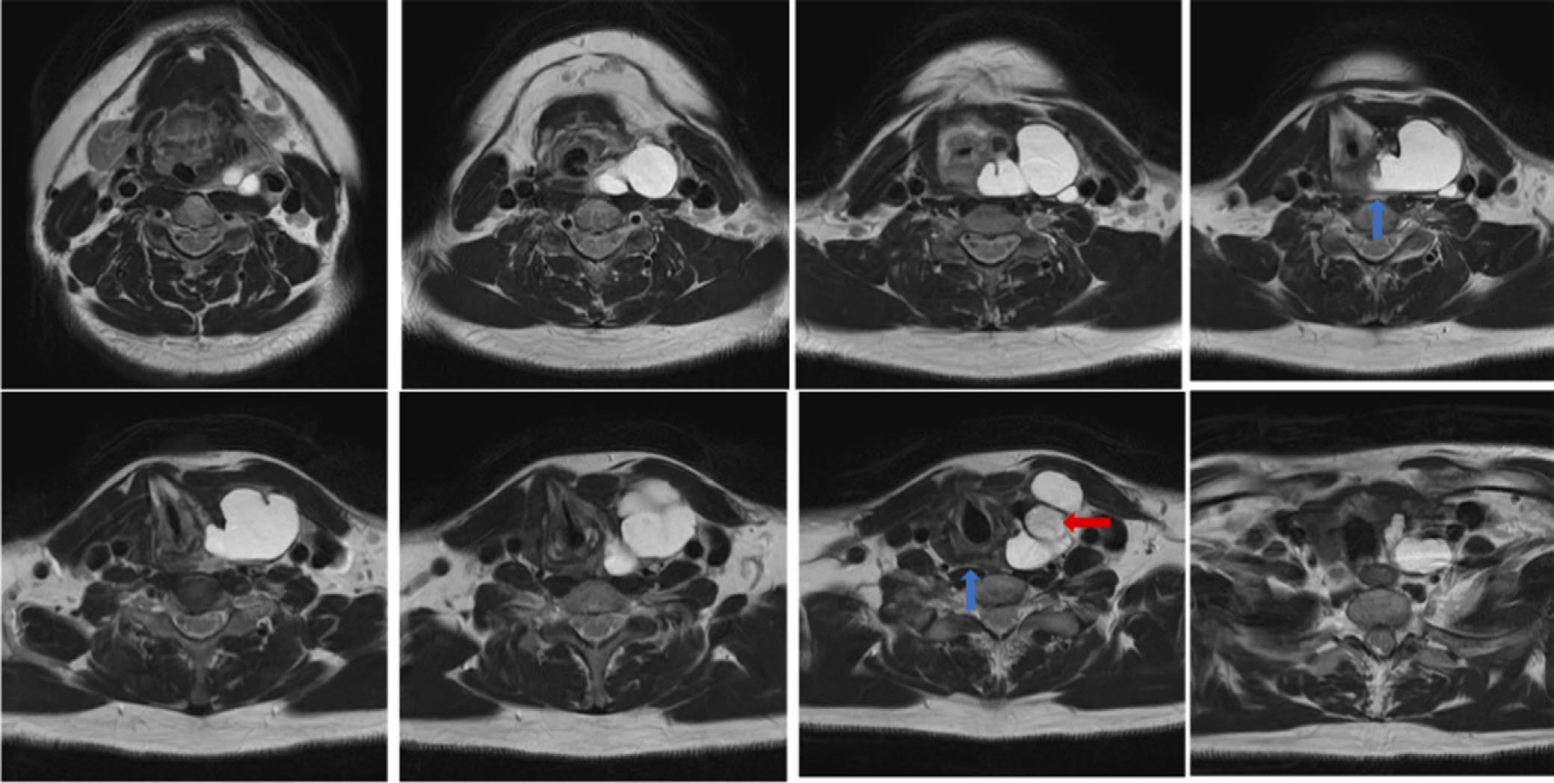
Axial MRI T1‐weighted images. The retropharyngeal space (RPS) was visualized as a linear layer of fat. The blue and red arrows indicate the RPS and primary tumor, respectively.

**FIGURE 3:**
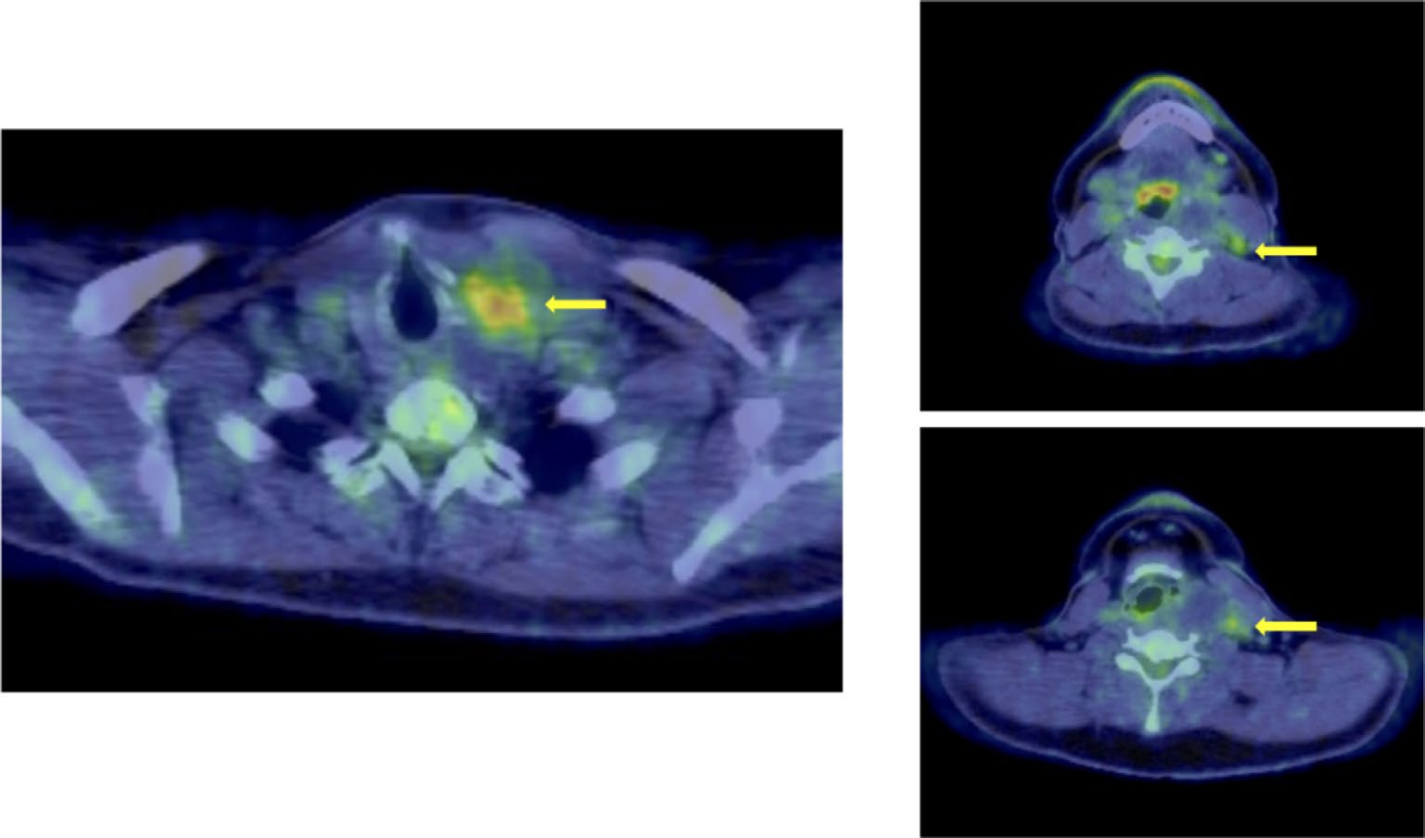
PET/CT images. Fluorodeoxyglucose (FDG) accumulation was observed in the left lobe of the thyroid gland and left cervical lymph nodes. The yellow arrow indicates FDG accumulation.

A 31‐year‐old male patient was referred to our department after a physical examination revealed neck swelling. He had no symptoms or compressive signs. His height was 173.6 cm, weight 99.1 kg, and BMI 32.9 kg/m^2^. A thyroid ultrasound was performed, but it was not possible to adequately evaluate due to his obesity. Clinical examination and magnetic resonance imaging (MRI) revealed a retropharyngeal goiter (Figures 1 and 2). The cystic lesion was located anterior to the retropharyngeal space (RPS), and the primary tumor in the left lobe was not in contact with the RPS (Figure 2). Papillary thyroid carcinoma (PTC) was diagnosed using fine‐needle aspiration cytology (FNAC) from the primary tumor. The thyroid function was euthyroid. Thyroglobulin was 51.1 ng/mL (reference range: 2.0–37.7 ng/mL), and anti‐thyroglobulin antibodies were negative. The clinical diagnosis was cT2N1bM0 PTC with retropharyngeal cystic goiter based on MRI, positron emission tomography/computed tomography (PET/CT) (Figure 3), CT, and FNAC.

A left neck dissection and total thyroidectomy were performed, and a tracheostomy was performed in anticipation of postoperative airway stenosis. The total operation time was 407 min, and the blood loss was 582 mL. Pathological examination revealed pT3N1b PTC with benign cystic lesions.

**FIGURE 4:**
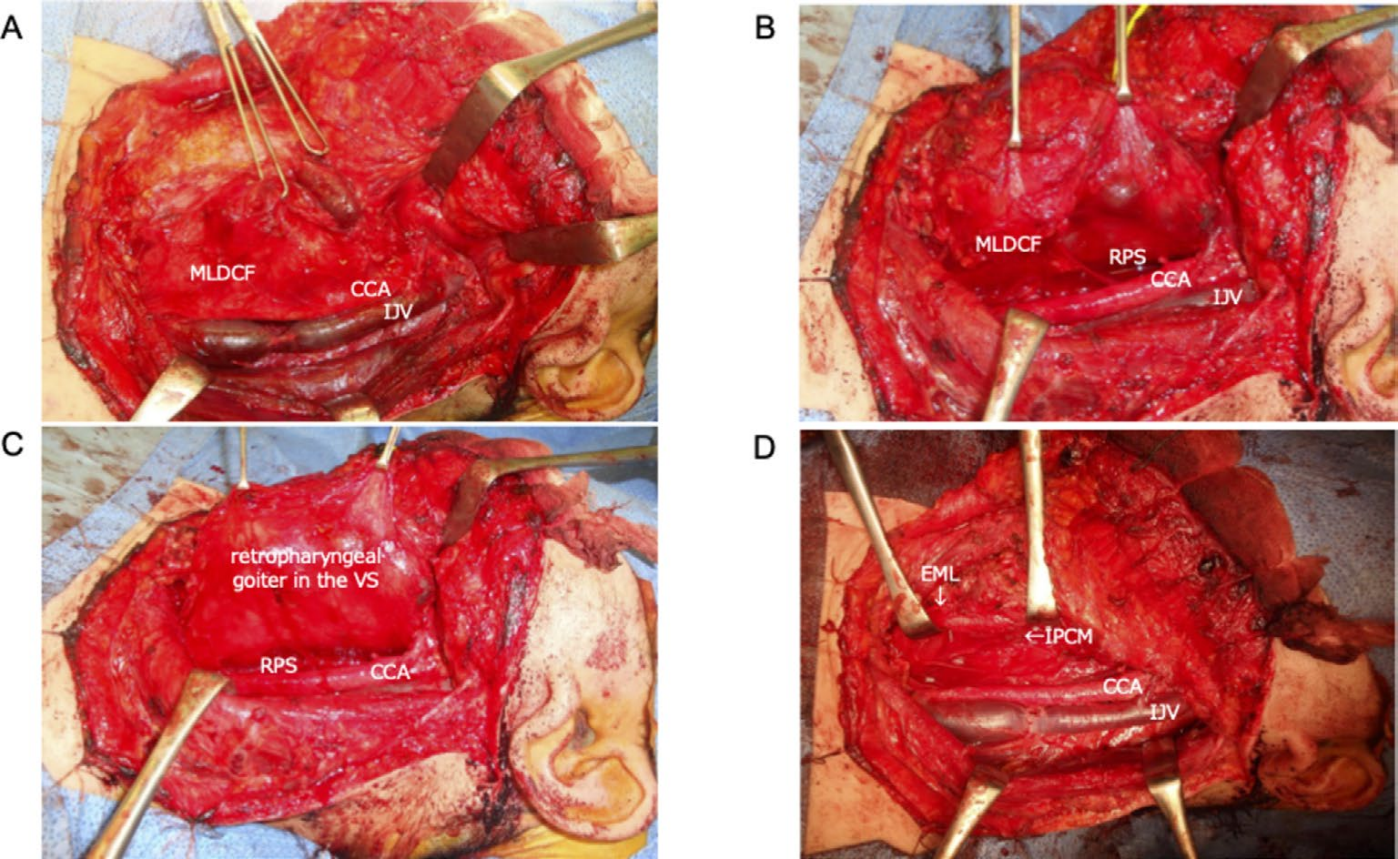
Intraoperative photographs. (A) After left neck dissection. (B) Opening of the retropharyngeal space. (C) The fully opened retropharyngeal space. (D) After total thyroidectomy. CCA, common carotid artery; EML, esophageal muscle layer; IJV, internal jugular vein; IPCM, inferior pharyngeal constrictor muscle; MLDCF, middle layer of the deep cervical fascia; VS, visceral space.

**FIGURE 5:**
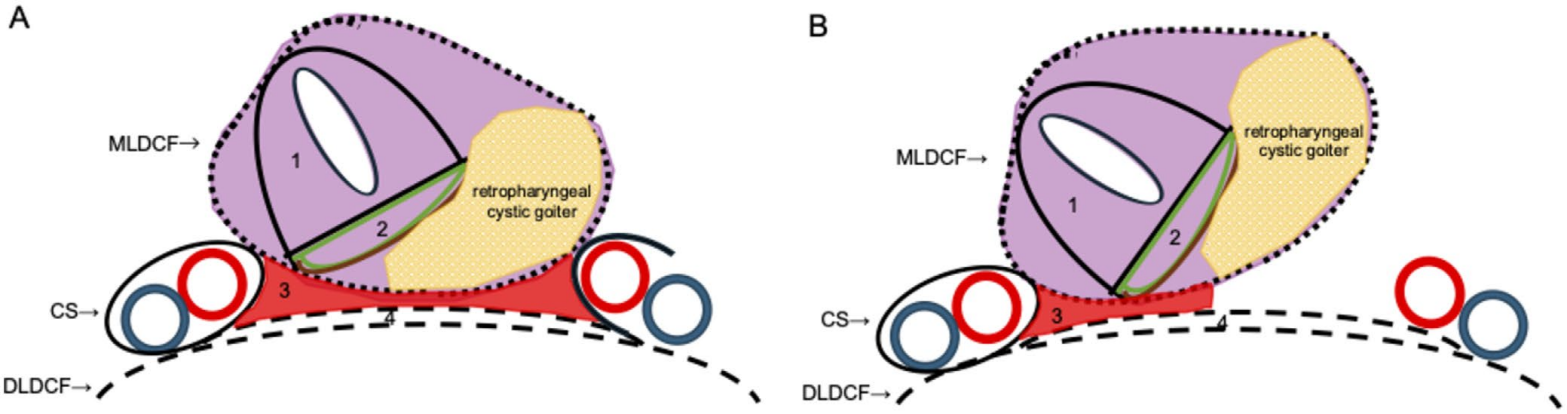
Schematic representations of the intraoperative findings. (A) After left neck dissection. (B) The opened retropharyngeal space. (1) Larynx, (2) Hypopharynx (green: Hypopharyngeal mucosa, brown: Pharyngeal muscles), (3) Retropharyngeal space, (4) Danger space. CS, carotid space; DLDCF, deep layer of the deep cervical fascia; MLDCF, middle layer of the deep cervical fascia. Dotted line: MLDCF, broken line: DLDCF, purple area: Visceral space, red area: Retropharyngeal space, red circle: Common carotid artery, blue circle: Internal jugular vein.

Intraoperative photographs and schematic representations were as follows: (1) after left neck dissection, the fascia between the visceral space (VS) and carotid space (CS) was preserved (Figures 4A and 5A); (2) the fascia between the VS and CS was incised to open the RPS (Figure 4B); (3) the RPS was open but the retropharyngeal goiter did not extend into the RPS. A retropharyngeal goiter was present in the VS (Figures 4C and 5B); (4) after the total thyroidectomy, the esophageal muscle layer and inferior pharyngeal constrictor muscle were exposed (Figure 4D).

After surgery, the patient was administered levothyroxine sodium 150 μg/day for hypothyroidism.

## 2 Discussion

The RPS is the potential space located posterior to the pharynx and esophagus, containing fat and lymph nodes. Previous reports have described retropharyngeal goiters of benign thyroid disease extending into the RPS [1]. We previously illustrated, using schematic representations, that a non‐invasive retropharyngeal goiter similar to this case did not extend into the RPS, but intraoperative photographs could not be provided at the time [2]. This report provides such intraoperative photographic evidence.

The thyroid gland is located within the VS and is enclosed by the middle layer of the deep cervical fascia (MLDCF). Retropharyngeal goiter typically grows within the VS, and when it enlarges superiorly, it can extend into the pharyngeal mucosal space (PMS) since the VS and PMS are continuous [2].

The MLDCF separates the VS from the RPS [2]. Hypopharyngeal carcinoma (HPC) arises within the VS. Even in advanced HPC, only about 10% of cases break through the MLDCF, extend into the RPS, and invade the prevertebral space [3]. Our findings indicate that the MLDCF prevents retropharyngeal goiters from extending into the RPS.

These findings provide important insights for surgeons who need to understand the relationship between the cervical spaces and retropharyngeal goiters.

## Author Contributions

**Masami Suzuki:** writing – original draft, writing – review and editing. **Naohiro Yoshida:** writing – review and editing. **Mikio Shimazaki:** writing – review and editing.

## Funding

The authors have nothing to report.

## Consent

Written and informed consent was obtained from the patient to publish his clinical health‐related information.

## Conflicts of Interest

The authors declare no conflicts of interest.

## Data Availability

Data sharing not applicable to this article as no datasets were generated or analyzed during the current study.
